# Transplacental transmission of field and rescued strains of BTV-2 and BTV-8 in experimentally infected sheep

**DOI:** 10.1186/1297-9716-44-75

**Published:** 2013-09-05

**Authors:** Lasse Dam Rasmussen, Giovanni Savini, Alessio Lorusso, Anna Bellacicco, Massimo Palmarini, Marco Caporale, Thomas Bruun Rasmussen, Graham J Belsham, Anette Bøtner

**Affiliations:** 1 National Veterinary Institute, Technical University of Denmark, 4771 Kalvehave, Lindholm, Denmark; 2 Istituto Zooprofilattico Sperimentale dell’Abruzzo e Molise “G. Caporale”, G. Caporale, via Campo Boario, Teramo 64100, Italy; 3 MRC Centre for Virus Research, Institute of Infection, Immunity and Inflammation, University of Glasgow, 464 Bearsden Road, Glasgow G61 1QH, Scotland, UK; 4 College of Medical, Veterinary and Life Sciences, University of Glasgow, 464 Bearsden Road, Glasgow G61 1QH, Scotland, UK

## Abstract

Transplacental transmission of bluetongue virus has been shown previously for the North European strain of serotype 8 (BTV-8) and for tissue culture or chicken egg-adapted vaccine strains but not for field strains of other serotypes. In this study, pregnant ewes (6 per group) were inoculated with either field or rescued strains of BTV-2 and BTV-8 in order to determine the ability of these viruses to cross the placental barrier. The field BTV-2 and BTV-8 strains was passaged once in Culicoides KC cells and once in mammalian cells. All virus inoculated sheep became infected and seroconverted against the different BTV strains used in this study. BTV RNA was detectable in the blood of all but two ewes for over 28 days but infectious virus could only be detected in the blood for a much shorter period. Interestingly, transplacental transmission of BTV-2 (both field and rescued strains) was demonstrated at high efficiency (6 out of 13 lambs born to BTV-2 infected ewes) while only 1 lamb of 12 born to BTV-8 infected ewes showed evidence of in utero infection. In addition, evidence for horizontal transmission of BTV-2 between ewes was observed. As expected, the parental BTV-2 and BTV-8 viruses and the viruses rescued by reverse genetics showed very similar properties to each other. This study showed, for the first time, that transplacental transmission of BTV-2, which had been minimally passaged in cell culture, can occur; hence such transmission might be more frequent than previously thought.

## Introduction

Bluetongue virus (BTV) is a member of the *Orbivirus* genus within the family *Reoviridae.* The virus has an RNA genome consisting of 10 separate double-stranded (ds) RNA segments each encoding at least one protein [1-4]. The virus particles consist of a multi-layered protein capsid enclosing the dsRNA segments. Some 26 different serotypes of BTV are known, including the Toggenburg virus [5] and a recently isolated virus from Kuwait [6]. The virus serotype is determined by segment 2, encoding VP2 [7,8]; this protein is exposed on the outer surface of the virus particle.

The virus can infect a variety of ruminant hosts including cattle, sheep and goats. In cattle, infection is normally sub-clinical but in sheep a variety of clinical signs can be observed including pyrexia, nasal discharge, salivation and facial oedema [2,9]. Initial replication of BTV in ruminants occurs within lymph nodes from where it is disseminated throughout the body. Viremia, defined as infectious virus in blood, can last for a significant period of time (≥ 11 days in sheep and many weeks in cattle, see [10]) even after the induction of neutralizing antibodies; the infectious virus is largely cell associated (see [9]). Furthermore, BTV RNA can be detected in the blood, by RT-PCR, for a significantly longer period of time (e.g. 111–222 days in cattle, see [10]) than infectious virus can be isolated [11]. It is generally only during the viremic period that insect vectors can ingest virus, become infected and subsequently transmit the virus to new ruminant hosts [9,10]. Transmission of BTV from one animal to another is normally achieved through the bites of *Culicoides* midges [9,12]. Following ingestion of a blood meal containing infectious virus, the virus replicates within the midge and it takes approximately 10 days before the virus can be transmitted efficiently to a new ruminant host [12]. Under certain circumstances, additional routes of virus transmission can be observed. There is some evidence for oral transmission of BTV-8 in calves, either through contact with an infected placenta or through ingestion of milk “spiked” with virus [13,14]. Similarly, contact transmission of BTV-26 between goats has been suggested [15] and infection with BTV-11 has been reported following ingestion of pooled colostrum in California where BTV is endemic [16]. More importantly, certain live attenuated BTV vaccine strains, passaged extensively in cell culture, were observed to be capable of transplacental transmission in both cattle and sheep [9,17,18] but early studies with wild-type (wt) BTV did not show this property (e.g. [19,20]). However, unexpectedly, following the introduction of the pathogenic BTV-8 into Northern Europe in 2006, it was observed that transplacental transmission of this virus strain occurred in both cattle and sheep [13,21-26]. A summary of the reported information on transplacental transmission of BTV in cattle and sheep has been published [27]. The particular features of the European BTV-8 virus which are responsible for transplacental transmission are not known. However, this property of the virus and the subsequent birth of viremic offspring may contribute towards its ability to be maintained from one year to the next (overwintering), within Northern Europe, in the absence of an active vector population during the winter months.

Recently it has become possible to rescue defined BTVs using a reverse genetics system [28] which relies on using specific RNA transcripts produced from cloned cDNA. The primary objective of this study was to characterize the properties, in sheep, of BTV-2 and BTV-8 (field strains) alongside the rescued BTV-2 and BTV-8 viruses derived from cloned cDNA. This should also determine whether the parental viruses (grown once in *Culicoides* cells (KC) and once in sheep choroid plexus (CP-TERT) cells) and the rescued viruses (inevitably involving further growth of the viruses in cell culture), displayed the same growth and transmission properties. In this study, pregnant ewes were inoculated with the selected BTV at about 1 month pre-term. All the inoculated ewes became viremic and seroconverted against BTV prior to parturition. The transplacental transmission of the viruses to the lambs was analyzed in each case.

## Materials and methods

### Viruses

BTV-2 wt was isolated from the spleen of an adult sheep infected during the 2001 Sardinian outbreak of bluetongue. This BTV-2 virus was isolated before the first vaccination campaign, with modified live vaccines (MLV), was initiated in 2002 (within Sardinia). Furthermore, this strain could be differentiated from the vaccine strain using a real time PCR system (as described and used previously [18,29]) and sequence analysis indicated that segments 5 and 10 of the viral RNA are 100% identical to the BTV-2/2000 Italy field strain and to other Mediterranean BTV-2 field strains while they differ (by 9% in Segment 5 and 17% in segment 10) from the BTV-2 South Africa MLV (G. Savini, unpublished results). The BTV-2 wt isolate was passaged once in KC cells and once in the CP-TERT cell line [30] prior to the experiment in order to amplify the virus to a concentration suitable for inoculation. The BTV-8 wt strain from The Netherlands (NET2006/04) was isolated from a sheep in 2006, passaged once in KC cells and once in BHK cells at the Institute for Animal Health, Pirbright, UK.

BTV-8 rescued by reverse genetics (termed BTV-8 rg in this study) has been described previously [4] and was derived from the BTV-8 NET2006/04 strain referred to as BTV-8 wt in this study. The BTV-2 strain rescued by reverse genetics (termed BTV-2 rg) was derived from an Italian strain isolated from an Italian sheep infected during the Sardinian outbreak in 2000. The isolate was originally passaged once in embryonated eggs, once in Vero cells and 3 times in BHK cells [31]. BTV-2 rg was rescued in BSR cells essentially as described previously [28] with slight modifications including a double transfection in which BSR cells were transfected initially with transcripts encoding VP1, VP3, VP4, NS1, VP6 and NS2 and then underwent a second transfection which included all 10 segments after 18 h incubation. Plaques were picked when visible by eye [4].

The virus stocks for each of the BTV strains were titrated in BSR cells just before use and were diluted to produce the inoculum with the following titres: BTV-2 wt (10^6.5^ TCID_50_/mL), BTV-2 rg (10^6.8^ TCID_50_/mL), BTV-8 wt (10^6.6^ TCID_50_/mL) and BTV-8 rg (10^6.7^ TCID_50_/mL).

### Animal inoculations

All experimental procedures and animal management protocols were carried out in accordance with the requirements of the Danish Animal Experimentation Inspectorate, license no. 2008/561-1541.

Twenty-eight pregnant sheep (Texel × Finuld), all born in 2010 after the termination of the Danish Bluetongue vaccination programme, were divided into four groups containing 6 sheep each and a control group containing 4 sheep. Prior to the experiment, all animals were tested and found to be negative for anti-BTV antibodies and virus. Each ewe was confirmed by ultrasound scanning to be carrying a single foetus. Animals in the four groups were inoculated sub-cutaneously on the inside of the thigh with 1 mL, containing approx. 10^6.7^ TCID_50_, of one of the four different virus strains to ensure infection of each animal. Four control ewes were inoculated in the same way with virus-free cell culture medium. The two groups of ewes inoculated with BTV-2 rg and BTV-8 rg were kept in separate boxes within stables approved for animal experiments using genetically modified organisms (GMO). The BTV-8 wt infected animals were housed in a separate stable, while the animals infected with BTV-2 wt and the control animals were housed within another stable but with the two groups in two individual pens that were separated by a walkway approximately 70 cm wide.

### Sampling

After virus inoculation, the ewes were monitored daily, for a period of 14 days, for the appearance of clinical signs and rectal temperatures were recorded. EDTA stabilised blood and untreated blood (for serum preparation) samples were collected at frequent intervals (0, 3, 6, 10, 14, 22 and 29 days post-inoculation (dpi)) in order to follow the presence of infectious BTV, BTV RNA and anti-BTV antibodies in the experimentally infected animals. Furthermore, EDTA stabilised blood and untreated blood samples were collected just after lambing and before the ewes were euthanized one week later.

After lambing, colostral milk samples were collected from all ewes by milking and on the following 6 days milk samples were collected daily. All milk samples were kept at 4 °C until analysed. Blood samples were collected from the lambs before the uptake of colostrum and again, 3 days post lambing (dpl) and when they were euthanized, generally at 7 dpl. A few lambs and the infected ewes were euthanized at 8–10 dpl but the results are shown as 7 dpl for clarity of the figures. Lambs from 3 ewes were euthanized soon after birth due to welfare reasons. All blood and serum samples were kept at 4 °C until analysis.

### Tissue collections

Tissue samples, from the spleen and mesenteric lymph nodes, were collected from all BTV- inoculated animals (sheep 5–28) at necropsy. From the lambs, brain tissue was also collected. Just after lambing, the cotyledons were collected from the placenta. Tissue samples were stored at −80 °C. For detection of BTV RNA, tissue samples were prepared by homogenization in PBS (or 0.9% NaCl) using a ratio of 1:10, the lysates were centrifuged at 3000 × *g* for 15 min and the supernatants were stored at −80 °C until analysis.

### Quantitation of infectious virus

Infectious BTV was measured within blood and milk samples by titration as described [32].

### Detection of BTV RNA

For the detection of BTV RNA, total nucleic acids were extracted from 100 μL of milk, EDTA blood or tissue supernatants using a MagNA pure LC Total Nucleic Acid Isolation Kit with a MagNA pure LC robot (Roche Diagnostics, Hvidovre, Denmark) and eluted in water (50 μL). Pan-BTV one-step quantitative RT-PCR (RT-qPCR) assays were performed using a previously described protocol [32-34] on the extracted nucleic acids using an M×3005p qPCR system (Agilent Technologies, Hørsholm, Denmark). BTV serotype-specific RT-qPCRs were performed using the BTV2G-IAH and BTV8G-IAH kits as described by the manufacturer (LSI, Lissieu, France).

### Anti-BTV antibody detection

Anti-BTV antibodies in serum were detected using ID Screen® Bluetongue Competition kit as described by the manufacturer (ID vet, Montpellier, France). The level of anti-BTV antibodies in milk samples was determined using the ID Screen® Blue Tongue Milk Indirect kit as described by the manufacturer (ID vet, Montpellier, France).

## Results

### Experimental infections

In order to examine the characteristics of the different BTV strains in sheep, a study was performed in which two sets of field and rescued viruses (BTV-2 and BTV-8) were inoculated into pregnant ewes at about 4 months of gestation (1 month prior to expected parturition). The majority of the lambs were born around 28 dpi (range was 17–38 days). Most (24/28) of the lambs were born healthy with the exception of 3 which had to be euthanized for welfare reasons (lambs 6, 16 and 25 from sheep infected with BTV-2 wt, BTV-8 wt and BTV-2 rg respectively) and one that was born dead (lamb 15 from a ewe infected with BTV-8 wt).

### Clinical signs in pregnant ewes after inoculation with BTV-2 or BTV-8

**Figure 1:**
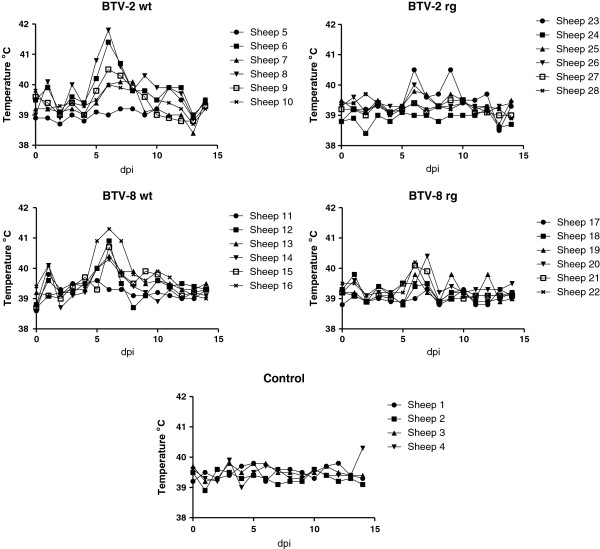
**Temperature responses in BTV infected ewes.** Pregnant ewes were inoculated with the indicated BTV (or none, control) on day 0. Rectal temperatures were recorded from each animal for the following 14 days as indicated.

Following virus inoculation, the ewes were monitored on a daily basis for clinical signs and clear evidence (see Figure 1) for pyrexia was apparent at 5–7 dpi in some, but not all, of the animals which were inoculated with BTV-2 wt and the BTV-8 wt. Weaker temperature responses were also seen in some animals inoculated with the BTV-2 rg and BTV-8 rg. Signs of respiratory distress and nasal discharge were also seen in all of the 6 ewes that received the BTV-2 wt. At 14 dpi, one of the control ewes (sheep 4) also had an elevated rectal temperature.

### Detection of BTV RNA in blood

**Figure 2:**
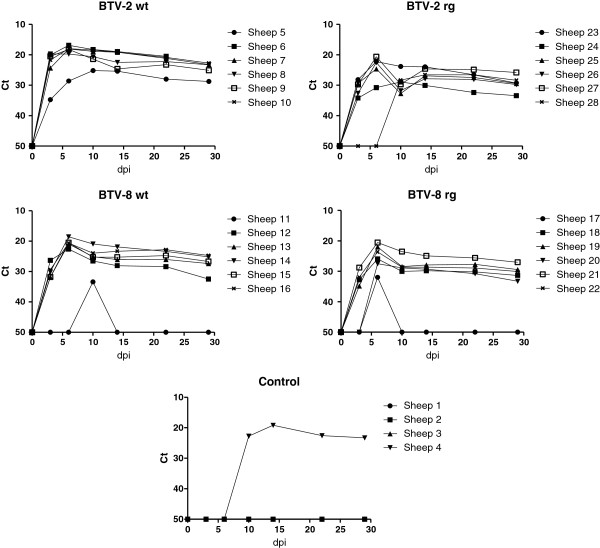
**BTV RNA, detected by RT-qPCR, in blood samples collected from ewes.** Pregnant ewes were infected on day 0 and blood samples were collected on the indicated days and assayed using a Pan-BTV RT-qPCR assay. Note, the presence of BTV RNA within sheep 4, which was one of the control animals (see text).

Blood samples were collected from each of the animals at 0, 3, 6, 10, 14, 22 and 29 dpi and analyzed for the presence of BTV RNA using RT-qPCR assays (Figure 2). In most (20/24) of the BTV-inoculated ewes, BTV RNA was observed in the blood at 3 dpi, the level of viral RNA increased further to peak levels in most animals at 6 dpi. Subsequently, the amount of BTV RNA in the blood of these ewes gradually declined through to 29 dpi although it was still readily detectable in each case. In ewe 20, inoculated with BTV-8 rg, BTV RNA was only detected from 6 dpi while in sheep 28, inoculated with BTV-2 rg, a further delayed appearance of BTV RNA in the blood was observed. BTV RNA was not detected at 3 or 6 dpi but was present at 10 dpi and subsequently. Furthermore, two ewes inoculated with BTV-8 wt (sheep 11) and BTV-8 rg (sheep 17) only had detectable levels of BTV RNA in the blood on a single sampling day (days 10 and 6 respectively) but the virus may have been present for a few days in each case (since sampling did not occur every day).

Samples collected at 14 dpi were analyzed using BTV serotype-specific RT-qPCR, and positive reactions, as anticipated, were only obtained with the expected serotype in each case (Table 1). Blood samples from sheep 11 and 17 were negative in these assays consistent with the failure of the pan-BTV assays to detect BTV RNA on this day in these two sheep.

**Table 1:** Serotype-specific RT-qPCR assays on blood samples at 14 dpi.

**Sheep**	**Inoculum**	**BTV-2**	**BTV-8**	**PanBTV**
1	No virus	-	-	-
2		-	-	-
3		-	-	-
4	*	23.01	-	19.15
5	BTV-2 wt	28.86	-	25.37
6		23.94	-	18.95
7		23.48	-	19.15
8		24.52	-	22.48
9		26.59	-	24.65
10		24.01	-	19.14
11	BTV-8 wt	-	-	-
12		-	32.92	28.08
13		-	29.5	26.09
14		-	25.8	21.9
15		-	30.36	25.24
16		-	26.31	23.33
17	BTV-8 rg	-	-	-
18		-	33.66	29.82
19		-	32.14	27.92
20		-	37.87	29.3
21		-	29.89	24.93
22		-	32.88	28.64
23	BTV-2 rg	27.84	-	23.96
24		31.82	-	30.06
25		28.41	-	26.91
26		30.53	-	27.87
27		27.45	-	24.63
28		28.3	-	26.55

* as indicated in text, this control ewe became infected with BTV-2.

Unexpectedly, one of the 4 negative control ewes (sheep 4) also had BTV RNA in its blood from 10 dpi onwards (Figure 2). Use of the serotype specific RT-qPCR assays with these samples indicated that the sheep had become infected with BTV-2 (see Table 1); it should be noted that the control animals were housed in the same airspace as the BTV-2 wt inoculated ewes but within separate pens (with a 70 cm wide passage) inside the animal accommodation. This infection is consistent with the pyrexia observed in this animal at 14 dpi (see above). The appearance of BTV RNA in the blood and the pyrexia were delayed by about 7 days relative to the inoculated animals and suggests direct transmission of BTV-2 from the inoculated animals.

### Viremia in BTV inoculated ewes

**Figure 3:**
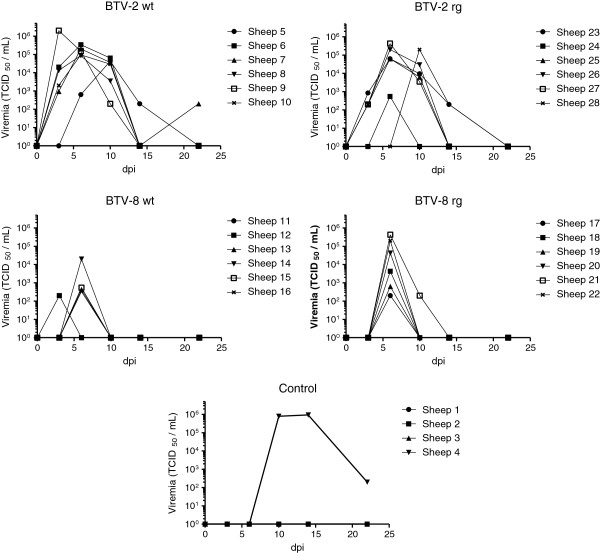
**Infectious BTV in blood samples of inoculated ewes.** Selected blood samples, collected on the indicated days, were assayed for the presence of infectious BTV by titration on cells.

The presence and level of infectious BTV were determined in blood samples taken from the inoculated ewes and are shown in Figure 3. In general, a higher level and longer duration of viremia was observed in sheep infected with BTV-2 than in those infected with BTV-8. Viremia was observed at 3 dpi in most of the sheep inoculated with each of the BTV-2 strains (except in sheep 5, 24 and 28) but just in one animal (sheep 12) inoculated with the BTV-8 wt. Furthermore, no viremia was detectable at 3 dpi in sheep which received BTV-8 rg although BTV RNA was detectable in the blood at this stage (see Figure 2), albeit at a fairly low level; this may be a reflection of the difference in sensitivity of the two different assays. At 6 dpi, infectious virus was detectable in the blood of all 4 groups of BTV-inoculated sheep and indeed the peak level of viremia was observed on this day for nearly all of the BTV-inoculated ewes. By 10 dpi, the level of BTV-8, had greatly declined whereas the titers of BTV-2 (both wt and rg strains) were still near peak levels in many animals (Figure 3) and indeed for sheep 28 this was the first day that BTV-2 rg appeared in the blood. However, by 14 dpi little or no infectious virus could be detected in the blood of most ewes although readily detectable levels of BTV RNA remained (see Figure 2). Sheep 4, (as shown in Figure 2) was also found to have infectious virus in its blood by 9 dpi (Figure 3) and this was maintained at high levels at 14 dpi, a result consistent with the unexpected detection of BTV RNA in one of the control animals. The duration and level of viremia in this animal was similar to that seen with the sheep inoculated with the BTV-2 wt but the development of the viremia was delayed (by about 7 days) relative to the inoculated sheep which is consistent with the delayed appearance of BTV in the blood (Figure 2) and the late pyrexia (Figure 1).

### Seroconversion against BTV

**Figure 4:**
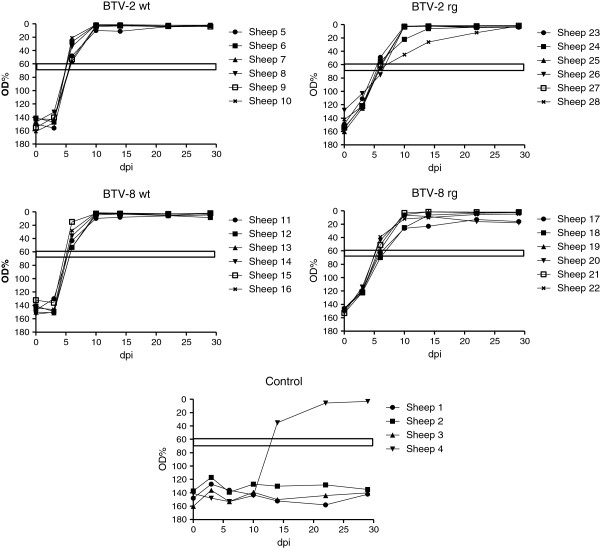
**Production of anti-BTV antibodies in ewes.** Serum samples collected on the indicated days from inoculated ewes were assayed for the presence of anti- BTV antibodies using an ELISA as described in Materials and methods. The horizontal bars indicate the boundary between positive and negative sera, values which fall within the bar are considered inconclusive.

Most (18/24) of the BTV-inoculated sheep had conclusively seroconverted against BTV by 6 dpi (as judged by ELISA) and the antibody levels in all the remaining animals had reached the diagnostic threshold level by 10 dpi (Figure 4). The presence of the anti-BTV antibodies in these animals was maintained up to the last samples analysed at 28 dpi. It is interesting to note that this included sheep 11 and 17 which only showed a short-lived presence of BTV RNA in the blood (see Figure 2). Comparison of the antibody responses indicated that the sheep reacted with different kinetics to the BTV-2 wt compared to the BTV-2 rg (Figure 4). At 3 dpi, the antibody responses to BTV-2 wt were lower than those observed with BTV-2 rg (*p* < 0.005) (although it should be noted that all of these antibody responses were still below the diagnostic threshold for the assay) whereas, in contrast, at 6 dpi, the clearly seropositive anti-BTV responses were higher (*p* < 0.01) in the BTV-2 wt inoculated ewes (6 out of 6 were seropositive) than in the ewes that received BTV-2 rg (only 2 out of these 6 were conclusively seropositive at 6 dpi). Thus, it appears that the antibody response, once initiated, was faster in the BTV-2 wt inoculated ewes. Analogous observations were also made in the animals that were inoculated with BTV-8 wt and rg viruses. At 3 dpi, there was no apparent response in BTV-8 wt infected animals but a small response (albeit below the diagnostic threshold) in the BTV-8 rg inoculated ewes. At 6 and 10 dpi, significantly higher (*p* < 0.01 and *p* < 0.05 respectively) antibody responses were detectable in the BTV-8 wt infected animals compared to the BTV-8 rg infected group (all the ewes inoculated with BTV-8 wt were seropositive at 6 dpi whereas only 4 out of 6 ewes inoculated with the BTV-8 rg were seropositive at this time). All the antibody responses were maintained throughout the rest of the experiment (Figure 4). Among the control animals, sheep 1, 2 and 3 remained seronegative throughout the experiment but sheep 4 had seroconverted by 14 dpi (but not by 10 dpi) consistent with the delayed infection with BTV-2 in this ewe described above (see also Figures 2 and 3).

### Transplacental transmission of BTV

**Figure 5:**
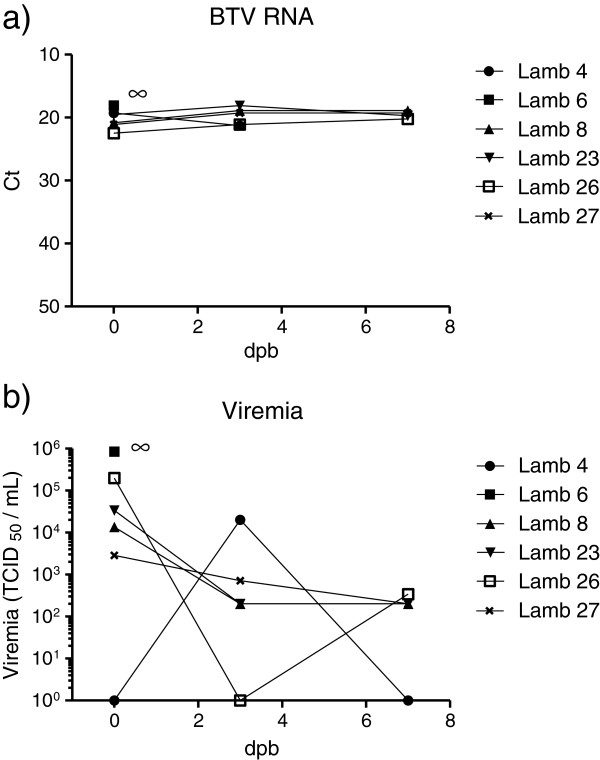
**Detection of BTV RNA and viremia in lambs.** Blood samples collected from lambs on the indicated days were assayed for the presence of BTV RNA **(panel a)** by RT-qPCR (as in Figure 2) and for infectious virus in cells **(panel b)** as for Figure 3. Note, lamb 6 was euthanized at birth (indicated by ∞).

To assess transplacental transmission of BTV from the ewes to the lambs, blood samples were collected from each lamb immediately after birth (before colostrum intake) and at 3 and 7 days of age (when possible). The presence of BTV RNA in these samples was assessed by RT-qPCR as above and the results are summarised in Table 2. In total, 6 lambs had BTV RNA detectable in their blood at birth. Two lambs, born to ewes 6 and 8 inoculated with BTV-2 wt, had high levels of BTV RNA in their blood at birth; this was maintained until euthanasia (Figure 5; but note that lamb 6 was euthanized on day 1). In addition, 3 lambs, born to sheep 23, 26 and 27 that had been inoculated with the BTV-2 rg, also had high levels of BTV RNA in their blood from birth which were maintained until euthanasia (ca. 7 days later). Finally, the control ewe (sheep 4), shown above to be infected with BTV-2, also produced a lamb with BTV RNA in its blood (Table 2 and Figure 5). In contrast, only 1 of the 12 lambs born to BTV-8 (wt or rg) infected ewes could be shown to carry BTV RNA at birth (note this was determined from organ samples (see below) since this lamb died at birth and blood samples were not collected). None of the other lambs born to BTV-8 inoculated ewes (either wt or rg strains) had BTV-8 in their blood (Table 2).

**Table 2:** Detection of BTV RNA by RT-qPCR in blood and tissues of lambs.

**Lamb**	** Virus**	**Blood sample**	**Blood sample**	**Blood sample**	**Spleen**	**Mesenteric lymph nodes**	**Brain**	**Cotyledons**
		**1**^**1**^	**2**^**2**^	**3**^**3**^				
1	None	-	-	-	-	-	-	-
2		-	-	-	-	-	-	-
3		-	-	-	-	-	-	-
4		19.32	21.3	18.5	24.86	29.71	27.28	N.D.
5	BTV-2 wt	-	-	-	-	-	-	28.93
6		18.08	N.D.	N.D.	21.87	26.83	24.98	23.54
7		-	-	-	-	-	-	N.D.
8		20.82	18.88	18.91	23.48	30.84	26.87	24.25
9		-	-	-	-	-	-	29.94
10		-	-	-	N.D.	N.D.	N.D.	25.96
11	BTV-8 wt	-	-	-	-	-	-	35.05
12		-	-	-	-	-	-	N.D.
13		-	-	-	-	-	-	28.49
14		-	-	-	-	-	-	27.65
15		N.D.^4^	N.D.^4^	N.D.^4^	19.99	27.41	25.96	22.72
16		-	-	N.D.	-	-	-	N.D.
17	BTV-8 rg	-	-	-	-	-	-	-
18		-	-	-	-	-	-	33.13
19		-	-	-	-	-	-	25.72
20		-	-	-	-	-	-	34.26
21		-	-	-	-	-	-	N.D.
22		-	-	-	-	-	-	26.61
23	BTV-2 rg	19.53	18.12	19.67	26.23	31.8	29.47	22.16
24		-	-	-	-	-	-	31.02
25		-	N.D.	N.D.	N.D.	-	-	N.D.
26		22.47	21.12	20.24	26.57	30.62	30.17	25.91
27		21.1	19.28	19.29	26.08	30.75	29.47	24.49
28		-	-	-	-	-	-	N.D.

1: Blood sample 1 was taken soon after birth before colostrum was received.

2: Blood sample 2 was normally taken at 3dpi but was delayed in some samples.

3: Blood sample 3 was normally taken at 7 dpi but was delayed in some samples.

4: Lamb 15 died at birth.

- indicates no Ct value obtained by 50 cycles.

*N.D.* = not determined.

Blood samples from the lambs shown to contain BTV RNA were also assayed for the presence of infectious BTV as described above. Five lambs were shown to be viremic at birth and lamb 4 was identified as viremic at 3 days of age (Figure 5). Lamb 6, which died shortly after birth, had the highest level of infectious BTV in its blood.

**Figure 6:**
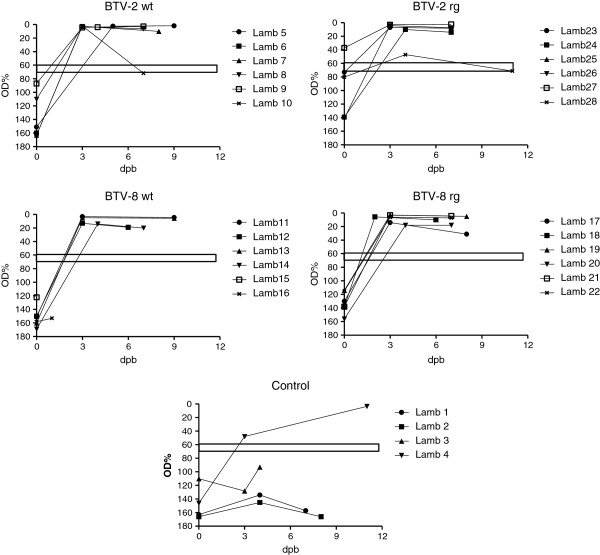
**Detection of anti-BTV antibodies in lambs.** Sera from lambs collected prior to ingestion of colostrum (at birth) and on the indicated days, subsequently, were assayed for the presence of anti-BTV antibodies by ELISA as in Figure 4.

The serum samples from the lambs were also assayed for the presence of anti-BTV antibodies by ELISA (see Figure 6). Only one animal (lamb 27, from a ewe inoculated with BTV-2 rg) had clearly seroconverted prior to birth although several others, from ewes infected with BTV-2, were close to the diagnostic cut off level. All of the other lambs born to the inoculated ewes (irrespective of virus strain) were shown to have seroconverted to BTV between birth and 3 days later (see Figure 6), presumably due to transfer of maternal antibodies in the colostrum. Indeed milk samples, collected on a daily basis after lambing, showed the presence of anti-BTV antibodies in the milk (see Table 3) from each of the infected ewes (including from the control sheep 4 which became infected with BTV-2). Three of the 13 BTV-2 infected ewes produced milk samples on the day of parturition which contained BTV RNA (Table 3) and two others produced milk that had low levels of BTV RNA on later days. In contrast, none of the sheep inoculated with BTV-8 had detectable BTV RNA in their milk on parturition day but one (sheep 16) had detectable BTV RNA on a later day. It should be noted that a high proportion (10 of 12) of these ewes still had BTV RNA in their blood (see Figure 2).This low proportion of BTV positive milk samples (and the low level of BTV RNA even in the positive samples) may be because the virus is closely associated with erythrocytes within the blood and hence not free to enter the milk, however this does not explain the apparent difference between the results from ewes inoculated with BTV-2 and BTV-8.

**Table 3:** Detection of BTV RNA and anti-BTV antibodies in tissues and milk from ewes.

**Sheep**	** Virus**	**Spleen**	**Mesenteric Lymph nodes**	**Milk (on day of parturition) **	**Anti-BTV abs in milk**
1	Control	N.D.	N.D.	N.D.	NEG
2		N.D.	N.D.	N.D.	NEG
3		N.D.	N.D.	N.D.	NEG
4		27.31	-	34.63	+
5	BTV-2 wt	30.07	-	-	+
6		23.14	31.03	29.76	+
7		24.13	33.37	-	+
8		26.36	-	-	+
9		27.59	31.6	-	+
10		N.D.	N.D.	N.D.	+
11	BTV-8 wt	33.00	-	-	+
12		28.81	32.03	-	+
13		28.56	35.10	-	+
14		30.42	34.12	-	+
15		30.59	33.04	-	+
16		30.13	32.65	−^1^	+
17	BTV-8 rg	35.51	31.99	-	+
18		29.66	-	-	+
19		28.32	33.98	-	+
20		29.84	-	-	+
21		26.19	-	-	+
22		28.31	32.86	-	+
23	BTV-2 rg	32.13	-	-	+
24		30.48	-	- ^1^	+
25		28.66	-	- ^1^	+
26		28.38	-	35.08	+
27		26.72	33.11	-	+
28		26.68	-	-	+

^1^: Milk samples collected on later days contained low levels of BTV RNA but it may be that the milk was contaminated by blood from the ewe.

### BTV RNA in ewe tissues

All spleen samples (except for sheep 10 which was lost), collected post-mortem from each of the BTV- inoculated ewes were found to contain BTV RNA as assessed by RT-qPCR (Table 3). Interestingly, BTV RNA was detected in the spleens of the 2 ewes (sheep 11 and 17) that only had a transient presence of BTV RNA in the blood, although the Ct values were relatively high (i.e. low levels of BTV RNA). This result appears consistent with the fact that these 2 sheep seroconverted to BTV (Figure 4). The spleen from ewe 4 (inadvertently infected with BTV-2) also contained BTV RNA (Table 3). In contrast, the mesenteric lymph nodes from only 12 of the 24 BTV- infected ewes contained detectable BTV RNA (Table 3).

### BTV RNA in tissue samples from lambs

Tissue samples from brain, spleen and mesenteric lymph nodes, collected post-mortem, from each of the lambs born to BTV-infected mothers or mock-infected controls were also tested for the presence of BTV RNA. As expected, each of the lambs that had BTV RNA in their blood (numbers 4, 6, 8, 23, 26 and 27; see Table 2), also had BTV RNA in these tissues. Since lamb 15 was found dead, no blood samples were collected but the spleen, lymph nodes and brain from this lamb were found to contain high levels of BTV RNA. In addition, the cotyledons (a portion of the placenta) were also tested for BTV RNA, when available, and found to be BTV positive in 17 of the 18 samples tested (Table 2), likely as a result of contamination from the blood of the ewes presumably during lambing. This was in accordance with the finding that the cotyledon samples from lambs that had BTV RNA in their own blood generally gave lower Ct values (i.e. higher levels of BTV RNA) compared to the other samples.

## Discussion

Each of the 4 virus strains (BTV-2 wt, BTV-2 rg, BTV-8 wt and BTV-8 rg) used in this study efficiently infected the inoculated pregnant ewes which all became viremic and, in most cases, the presence of viral RNA in the blood was maintained for an extended period (>25 days) including the time of parturition (Figures 2 and 3). In addition, all of the infected ewes seroconverted against the virus (Figure 4). A major focus of this study has been the transplacental transmission of the different viruses and this will be discussed in detail below. In addition, the unexpected horizontal transmission of BTV-2 is considered. The results show that the wt and rg strains of each serotype had very similar characteristics to each other but the BTV-2 and BTV-8 viruses show some differences. It was noted that BTV-2 wt produced more marked clinical signs (including respiratory distress and nasal discharge) than any of the other viruses and both the BTV-2 wt and the BTV-8 wt viruses produced more marked pyrexia than the rescued (rg) viruses. However, the significance of this is not clear since all animals acquired BTV RNA in their blood and seroconverted against the virus.

### Transplacental transmission of BTV-2 and BTV-8 in sheep

As described previously [27], transplacental transmission, in both cattle and sheep, is a documented property of the North European BTV-8 strain and also of BTV modified live vaccines or laboratory adapted strains that have been extensively passaged in eggs or tissue culture [9,17,18]. In contrast, earlier work with other field strains of BTV (not including BTV-2) did not show this property [19,20,27].

In this study we now show that a field strain of BTV-2, minimally passaged in tissue culture, is able to cross the placental barrier in experimentally infected ewes. The BTV-2 wt virus [31] had been passaged only once in KC cells and once in a sheep cell line and it is, therefore not expected to have changed its phenotype due to mutations accumulated after extensive cell culture passaging as shown for tissue culture adapted or vaccine strains. Both BTV-2 wt and BTV-2 rg were capable of transplacental transmission in approximately 50% of the infected ewes. Interestingly, transplacental transmission of BTV-2 occurred even in the negative control ewe (sheep 4) that had been infected, inadvertently, during co-housing with the experimentally infected sheep. Thus, the transplacental transmission of BTV-2 does not appear to be an artifact of the experimental inoculation procedure *per se* (i.e. using sub-cutaneous inoculation of high levels of virus).

There have been no previous reports on the transplacental transmission of field strains of BTV-2 but the clear detection of viremia in the lambs as well as the presence of BTV RNA in blood and tissue samples clearly demonstrate this process for both the BTV-2 wt and BTV-2 rg strains used in this study (Table 2 and Figure 5). Recently, it has been reported that an egg and cell culture-adapted strain of BTV-2 (used as a modified live vaccine) is able to cross the placenta of sheep and result in infection of foetuses [18]. It remains to be determined whether BTV-2, which has only been passaged within animals and transmitted by transfer of blood, is also able to cross the placental barrier. The BTV-2 wt strain used in the current study was isolated from a BTV-infected sheep in Sardinia prior to the use of modified live vaccine strains (from South Africa) on this island. In addition, it has been shown, using RT-qPCR assays [29] and genome sequencing, that this BTV-2 wt strain is distinct from the BTV-2 vaccine strain (G. Savini, unpublished results).

Transplacental transmission of the BTV-8 wt was observed at a lower frequency than expected, in only 1 of the 6 ewes, and in none of the ewes infected with the BTV-8 rg. Previous studies have indicated that transplacental transmission of BTV-8 wt has occurred in sheep [23,24] and cattle [20,35,36] but the frequency can be quite variable varying from 0-69% in sheep [21,24,25] and 20-35% in cattle [22,26,36]. This may depend, in part, on the time of infection in relation to the gestation period. It should be noted that BTV infection of ewes during the early stages of gestation (5^th^ and 6^th^ week) can have serious neurological consequences for the lambs (see [9,18]) when transplacental transmission (e.g. with vaccine strains) occurs. However, studies with an attenuated BTV-23 virus (passaged 20 times in Vero cells) in Merino sheep [37] showed that vaccination with this live virus (which produced no clinical disease) during the late stage of gestation had no apparent effect on the production of lambs despite causing high losses in the first and second thirds of the pregnancy. These data are consistent with the results observed here using infection with both the BTV-2 and BTV-8 strains since no abnormalities were observed in any of the lambs.

Each of the BTVs tested clearly infected the virus inoculated sheep since all had infectious virus and BTV RNA detectable in the blood and all of the inoculated animals seroconverted against BTV. The serotype specific RT-qPCR assays confirmed that the animals had indeed been infected with the serotype of BTV used for their inoculation (Table 1). At the time of birth, only one of the lambs (number 27) had conclusively seroconverted against BTV but all lambs from BTV-infected ewes rapidly seroconverted following ingestion of colostrum. These results were consistent with previous studies indicating that the presence of anti-BTV antibodies in lambs depends on the time point during gestation at which the BTV infection occurred, when the infection is late (as in this study) then lambs can be born viremic with or without anti-BTV antibodies [9,24]. It was apparent that the severity of clinical signs (elevated temperature and respiratory distress) together with the level and period of viremia (when infectious virus can be isolated) was much greater in the BTV-2 inoculated sheep than in the BTV-8 infected sheep. It may be that these features are relevant to the probability of transplacental transmission. However, it is noteworthy that similar levels and maintenance of BTV RNA in the blood, as measured by RT-qPCR, were observed for all of the viruses tested and each virus also induced seroconversion. Thus the level and maintenance of BTV RNA within the blood are clearly not indicative of the ability of BTV to cross the placenta.

There has been some discussion previously about the possible role of co-infection of BTV with pestiviruses (e.g. bovine viral diarrhea virus (BVDV) and border disease virus (BDV)) in transplacental transmission. Backx et al. [14] found a possible association between the timing of seroconversion to BVDV in a single dam and the production of a BTV positive calf. However, Zanella et al. [35] showed that 128 (16%) fetuses/calves analyzed in their study were BTV-8 positive but only 2 out of the 763 calves from dams that were tested were found to be coinfected with BTV-8 and BVDV while 9 fetuses/calves were exclusively BVDV positive. Thus these authors concluded that “BVDV did not play an important role as a cross-barrier enhancer”. We are unaware of any studies demonstrating a link between pestivirus infection and BTV transplacental transmission in sheep. In Denmark, the sheep are free from BDV and BVDV infection and, indeed, all sera from each of the ewes were seronegative in a BVDV ELISA (which cross reacts with antibodies against other pestiviruses) at the start of the experiment (day 0). For one group of animals (sheep 5–10), inoculated with BTV-2 wt, it has been shown that these animals seroconverted against pestiviruses by 29 dpi (data not shown). Based on a set of differentiating neutralization tests, it appears that the antibodies detected in these 6 animals were generated against BDV rather than BVDV. All of the other ewes remained seronegative for pestiviruses. Thus it seems that the BTV-2 wt inoculum was contaminated with BDV. We do not believe this has influenced the results significantly for several reasons. As indicated above, sheep 4, one of the control sheep, unexpectedly became infected with BTV-2 without receiving the same inoculum, however, this ewe did not seroconvert against pestiviruses but produced a BTV positive lamb (Table 2). Furthermore, 50% of the BTV-2 rg inoculated group (as with the BTV-2 wt group) also produced BTV infected lambs but none of these animals seroconverted against pestiviruses (data not shown). Thus there was no apparent linkage between pestivirus infection and BTV-2 transplacental transmission in this study.

### Oral transmission of BTV-2 in sheep?

An interesting feature of this study was the observation that one ewe (sheep 4), which was in the control group, became infected with BTV-2 (see Figures 2, 3 and 4). This sheep shared the same airspace with the sheep inoculated with BTV-2 wt but was in a different pen separated by a 70 cm wide corridor. The infection in sheep 4 was delayed by approximately one week compared to the directly inoculated animals and thus it appears that transmission of the virus has occurred between the infected animals and this control animal. The one week time delay does not seem sufficient for this transmission to have occurred through midge vectors since it takes approximately 2 weeks for ingested virus to replicate sufficiently within midges to enable efficient transmission to occur [12]. Furthermore, the animal isolation facilities should not allow access to midges but their introduction with the sheep, or maybe straw, cannot be completely excluded although the experiment was performed outside of the normal vector season within Denmark. It seems more likely that transmission occurred by oral ingestion of virus, e.g. on straw contaminated either during the inoculation procedure of the test group (by leaking of inoculum from inoculation site) or from virus shed during the early stages of infection and transferred, inadvertently, to the control group. Previous studies have also provided some evidence that transmission of BTV can occur between cattle and goats independently from midges [13-16] and it seems that this has occurred by oral transmission. It is also interesting to note that sheep 1, which did not show any evidence for being infected with BTV during the course of the studies presented here, had BTV RNA in its blood at 16 days after lambing of sheep 4, and in organs at autopsy, during a follow-up study (note the control animals, 1–4, were not euthanized with the sheep 5–28). Thus sheep 1 may also have become infected through oral transmission of the virus (likely via sheep 4 since the placenta of the lamb born to this ewe was not found in the pen). Infection via contact with an infected placenta has been described previously [13]. It is clear that a more focused analysis of oral transmission of BTV would be useful.

## Conclusions

This study shows that an Italian strain of BTV-2 minimally passaged in tissue culture (once in *Culicoides* cells and once in sheep cells) is able to cross the placental barrier in infected sheep. Although theoretically possible, it seems unlikely that the minimum passage of the virus in cells that are derived from a host species for BTV could change its phenotype in such a dramatic fashion. Thus, transplacental transmission, at least for this strain of BTV-2, might be more common than previously recognized and one of the mechanisms by which this virus overwinters when insect vector activity is very low.

## Competing interests

The authors declare that they have no competing interests.

## Authors’ contributions

LDR: supervised and performed the analysis of all samples, helped to draft the manuscript. GS: contributed to the experimental design, provided virus samples, assisted in drafting the manuscript. AL: provided virus samples and characterised them. ABe: Determined viraemia. MP: contributed to the experimental design, provided virus samples, assisted in drafting the manuscript. MC: characterized and provided virus samples. TBR: contributed to data analysis and drafting of the manuscript. GJB: contributed to the experimental design and data analysis, drafted the manuscript. AB: overall coordination of the project; contributed to the experimental design and planning of animal experimentation; helped to draft the manuscript. All authors read and approved the final manuscript.

## References

[B1] Roy P Orbivirus structure and assembly Virology 1996 44 1 11 10.1006/viro.1996.0028 8614976

[B2] Schwartz-Cornil I Mertens PP Contreras V Hemati B Pascale F Bréard E Mellor PS Maclachlan NJ Zientara S Bluetongue virus: virology, pathogenesis and immunity Vet Res 2008 44 46 10.1051/vetres:2008023 18495078

[B3] Wade-Evans AM Mertens PP Belsham GJ Sequence of genome segment 9 of bluetongue virus (serotype 1, South Africa) and expression analysis demonstrating that different forms of VP6 are derived from initiation of protein synthesis at two distinct sites J Gen Virol 1992 44 3023 3026 10.1099/0022-1317-73-11-3023 1331303

[B4] Ratinier M Caporale M Golder M Franzoni G Allan K Nunes SF Armezzani A Bayoumy A Rixon F Shaw A Palmarini M Identification and characterization of a novel non-structural protein of bluetongue virus PLoS Pathog 2011 44 e1002477 10.1371/journal.ppat.1002477 22241985 PMC3248566

[B5] Hofmann MA Renzullo S Mader M Chaignat V Worwa G Thuer B Genetic characterization of Toggenburg orbivirus, a new bluetongue virus, from goats, Switzerland Emerg Infect Dis 2008 44 1855 1861 10.3201/eid1412.080818 19046507 PMC2634640

[B6] Maan S Maan NS Nomikou K Veronesi E Bachanek-Bankowska K Belaganahalli MN Attoui H Mertens PP Complete genome characterisation of a novel 26th bluetongue virus serotype from Kuwait PLoS One 2011 44 e26147 10.1371/journal.pone.0026147 22031822 PMC3198726

[B7] Mertens PP Pedley S Cowley J Burroughs JN Corteyn AH Jeggo MH Jennings DM Gorman BM Analysis of the roles of bluetongue virus outer capsid proteins VP2 and VP5 in determination of virus serotype Virology 1989 44 561 565 10.1016/0042-6822(89)90447-9 2543130

[B8] Shaw AE Ratinier M Nunes SF Nomikou K Caporale M Golder M Allan K Hamers C Hudelet P Zientara S Breard E Mertens P Palmarini M Reassortment between two serologically unrelated bluetongue virus strains is flexible and can involve any genome segment J Virol 2013 44 543 557 10.1128/JVI.02266-12 23097432 PMC3536370

[B9] Maclachlan NJ Drew CP Darpel KE Worwa G The pathology and pathogenesis of bluetongue J Comp Pathol 2009 44 1 16 10.1016/j.jcpa.2009.04.003 19476953

[B10] Bonneau KR DeMaula CD Mullens BA Maclachlan NJ Duration of viraemia infectious to Culicoides sonorensis in bluetongue virus-infected cattle and sheep Vet Microbiol 2002 44 115 125 10.1016/S0378-1135(02)00106-2 12135632

[B11] Di-Gialleonardo L Migliaccio P Teodori L Savini G The length of BTV-8 viraemia in cattle according to infection doses and diagnostic techniques Res Vet Sci 2011 44 316 320 10.1016/j.rvsc.2010.12.017 21324498

[B12] Mellor PS Replication of arboviruses in insect vectors J Comp Pathol 2000 44 231 247 10.1053/jcpa.2000.0434 11041993

[B13] Menzies FD McCullough SJ McKeown IM Forster JL Jess S Batten C Murchie AK Gloster J Fallows JG Pelgrim W Mellor PS Oura CA Evidence for transplacental and contact transmission of bluetongue virus in cattle Vet Rec 2008 44 203 209 10.1136/vr.163.7.203 18708653

[B14] Backx A Heutink R Van-Rooij E Van-Rijn P Transplacental and oral transmission of wild-type bluetongue virus serotype 8 in cattle after experimental infection Vet Microbiol 2009 44 235 243 10.1016/j.vetmic.2009.04.003 19419822

[B15] Batten CA Henstock MR Steedman HM Waddington S Edwards L Oura CA Bluetongue virus serotype 26: infection kinetics, pathogenesis and possible contact transmission in goats Vet Microbiol 2013 44 62 67 10.1016/j.vetmic.2012.08.014 22986055

[B16] Mayo CE Crossley BM Hietala SK Gardiner IA Breitmeyer RE Maclachlan NJ Colostral transmission of bluetongue virus nucleic acid among newborn dairy calves in California Transbound Emerg Dis 2010 44 277 281 20557494 10.1111/j.1865-1682.2010.01149.x PMC2908191

[B17] Gibbs EP Lawman MJ Herniman KA Preliminary observations on transplacental infection of bluetongue virus in sheep-a possible overwintering mechanism Res Vet Sci 1979 44 118 120 228365

[B18] Savini G Lorusso A Paladini C Migliaccio P Di-Gennaro A Di-Provvido A Scacchia M Monaco F Bluetongue serotype 2 and 9 modified live vaccine viruses as causative agents of abortion in livestock: a retrospective analysis in Italy Transbound Emerg Disin press [doi:10.1111/tbed.12004] 10.1111/tbed.12004 22937914

[B19] Acree JA Echternkamp SE Kappes SM Luedke AJ Holbrook FR Pearson JE Ross GS Failure of embryos from bluetongue infected cattle to transmit virus to susceptible recipients or their offspring Theriogenology 1991 44 689 697 10.1016/0093-691X(91)90406-4 16727038

[B20] Roeder PL Taylor WP Roberts DH Wood L Jeggo MH Gard GP Corteyn M Graham S Failure to establish congenital bluetongue virus infection by infecting cows in early pregnancy Vet Rec 1991 44 301 304 10.1136/vr.128.13.301 1852081

[B21] De-Clercq K De-Leeuw I Verheyden B Vandemeulebroucke E Vanbinst T Herr C Méroc E Bertels G Steurbaut N Miry C De-Bleecker K Maquet G Bughin J Saulmont M Lebrun M Sustronck B De-Deken R Hooyberghs J Houdart P Raemaekers M Mintiens K Kerkhofs P Goris N Vandenbussche F Transplacental infection and apparently immunotolerance induced by a wild-type bluetongue virus serotype 8 natural infection Transbound Emerg Dis 2008 44 352 359 10.1111/j.1865-1682.2008.01044.x 18673339

[B22] Darpel KE Batten CA Veronesi E Williamson S Anderson P Dennison M Clifford S Smith C Philips L Bidewell C Bachanek-Bankowska K Sanders A Bin-Tarif A Wilson AJ Gubbins S Mertens PP Oura CA Mellor PS Transplacental transmission of bluetongue virus 8 in cattle, UK Emerg Infect Dis 2009 44 2025 2028 10.3201/eid1512.090788 19961692 PMC3044536

[B23] Worwa G Hilbe M Ehrensperger F Chaignat V Hofmann MA Griot C Maclachlan NJ Thuer B Experimental transplacental infection of sheep with bluetongue virus serotype 8 Vet Rec 2009 44 499 500 10.1136/vr.164.16.499 19377090

[B24] Saegerman C Bolkaerts B Baricalla C Raes M Wiggers L De-Leeuw I Vandenbussche F Zimmer JY Haubruge E Cassart D De-Clercq K Kirschvink N The impact of naturally-occurring, trans-placental bluetongue virus serotype-8 infection on reproductive performance in sheep Vet J 2011 44 72 80 10.1016/j.tvjl.2009.11.012 20061168

[B25] van der-Sluijs M Timmermans M Moulin V Noordegraaf CV Vrijenhoek M Debyser I De-Smit AJ Moormann R Transplacental transmission of Bluetongue virus serotype 8 in ewes in early and mid gestation Vet Microbiol 2011 44 113 125 10.1016/j.vetmic.2010.11.002 21145670

[B26] van der-Sluijs MT Schroer-Joosten DP Fid-Fourkour A Vrijenhoek MP Debyser I Gregg DA Dufe DM Moulin V Moormann RJ De-Smit AJ Effect of vaccination with an inactivated vaccine on transplacental transmission of BTV-8 in midterm pregnant ewes and heifers Vaccine 2012 44 647 655 10.1016/j.vaccine.2011.10.106 22107846

[B27] EFSA Panel on Animal Health and Welfare (AHAW) Scientific Opinion on bluetongue serotype 8 EFSA J 2011 44 2189 doi:10.2903/j.efsa.2011.2189. Available online at [http://www.efsa.europa.eu/efsajournal.htm]

[B28] Boyce M Celma CC Roy P Development of reverse genetics systems for bluetongue virus: recovery of infectious virus from synthetic RNA transcripts J Virol 2008 44 8339 8348 10.1128/JVI.00808-08 18562540 PMC2519640

[B29] Elia G Savini G Decaro N Martella V Teodori L Casaccia C Di-Gialleonardo L Lorusso E Caporale V Buonavoglia C Use of real-time RT-PCR as a rapid molecular approach for differentiation of field and vaccine strains of bluetongue virus serotypes 2 and 9 Mol Cell Probes 2008 44 38 46 10.1016/j.mcp.2007.06.005 17693055

[B30] Arnaud F Black S Murphy L Griffiths D Neil S Spencer TE Palmarini M The interplay between ovine Bst-2/Tetherin and endogenous retroviruses J Virol 2010 44 4415 4425 10.1128/JVI.00029-10 20181686 PMC2863748

[B31] Caporale M Wash R Pini A Savini G Franchi P Golder M Patterson-Kane J Mertens P Di-Gialleonardo L Armillotta G Lelli R Kellam P Palmarini M Determinants of bluetongue virus virulence in murine models of disease J Virol 2011 44 11479 11489 10.1128/JVI.05226-11 21865388 PMC3194974

[B32] OIE World Organisation for Animal Health (Office International des Épizooties: OIE) Manual of diagnostic tests and vaccines for terrestrial animals OIE, Paris, Bluetongue, Chapter 2.1.3 2009 [http://web.oie.int/eng/normes/MMANUAL/A_Index.htm]

[B33] Rasmussen LD Rasmussen TB Belsham GJ Strandbygaard B Bøtner A Bluetongue in Denmark during 2008 Vet Rec 2010 44 714 718 10.1136/vr.b4847 20525947

[B34] Shaw AE Monaghan P Alpar HO Anthony S Darpel KE Batten CA Guercio A Alimena G Vitale M Bankowska K Carpenter S Jones H Oura CA King DP Elliott H Mellor PS Mertens PP Development and initial evaluation of a real-time RT-PCR assay to detect bluetongue virus genome segment 1 J Virol Methods 2007 44 115 126 10.1016/j.jviromet.2007.05.014 17586061

[B35] Zanella G Durand B Sellal E Breard E Sailleau C Zientara S Batten CA Mathevet P Audeval C Bluetongue virus serotype 8: abortion and transplacental transmission in cattle in the Burgundy region, France, 2008–2009 Theriogenology 2012 44 65 72 10.1016/j.theriogenology.2011.07.015 21872306

[B36] Desmecht D Bergh RV Sartelet A Leclerc M Mignot C Misse F Sudraud C Berthemin S Jolly S Mousset B Linden A Coignoul F Cassart D Evidence for transplacental transmission of the current wild-type strain of bluetongue virus serotype 8 in cattle Vet Rec 2008 44 50 52 10.1136/vr.163.2.50 18621997

[B37] Flanagan M Johnson SJ The effects of vaccination of Merino ewes with an attenuated Australian bluetongue virus serotype 23 at different stages of gestation Aust Vet J 1995 44 455 457 10.1111/j.1751-0813.1995.tb03488.x 8825310

